# The IJHPR publishes its 100^th^ article, and other momentous milestones

**DOI:** 10.1186/2045-4015-2-48

**Published:** 2013-12-19

**Authors:** Bruce Rosen, Avi Israeli

**Affiliations:** 1 Myers-JDC-Brookdale Institute, POB 3886, Jerusalem 91037, Israel; 2 Hadassah–Hebrew University Medical Center, POB 12000, Jerusalem 91120, Israel; 3 Ministry of Health, Ben Tabai 2, Jerusalem 93591, Israel

## Abstract

The Israel Journal of Health Policy Research (IJHPR) was launched in January 2012 and it is now publishing its 100^th^ article. It was accepted into PubMed after only six months of publication and it has now also been accepted by Thomson Reuters for inclusion in the Web of Science as well as the Social Science Citation Index. It is rare for a new journal to reach these milestones at such an early stage in its development.

One of the key factors in the journal’s acceptance into these prestigious databases has been its unique national/international approach – exploring both what Israel can learn from health systems in other countries and what other countries can learn from Israeli health care. Another key factor has been its ability to attract high quality contributions from virtually all of the Israeli universities and research centers involved in health policy. A third important factor has been the journal’s ability to engage leading international scholars as contributors and/or editorial board members.

## 

The Israel Journal of Health Policy Research is now completing its second year of publication. We are pleased to take this opportunity to report on the progress of the IJHPR, to reflect on one of the journal’s unique roles, to share some of our plans for the year ahead, and to reiterate some of the ongoing challenges in cross-national learning.

## Progress report

The Israel Journal of Health Policy Research (IJHPR) was launched in January 2012 and it is now publishing its 100^th^ article. We are gratified that the journal was accepted into PubMed after only six months of publication and that it has now also been accepted by Thomson Reuters for inclusion in the Web of Science as well as the Social Science Citation Index. It is rare for a new journal to reach these milestones at such an early stage in its development.

One of the key factors in the journal’s acceptance into these prestigious databases has been its unique national/international approach – exploring both what Israel can learn from health systems in other countries and what other countries can learn from Israeli health care. Another key factor has been its ability to attract high quality contributions from virtually all of the Israeli universities and research centers involved in health policy. A third important factor has been the journal’s ability to engage leading international scholars as contributors and/or editorial board members.

This end-of-the-year editorial is a natural opportunity to thank all those who have contributed to the journal’s success. These include, but are not limited to, the Israel National Institute for Health Policy (the journal’s sponsor), BioMed Central (the journal’s publisher), the editorial board, and our many contributors, reviewers and readers from around the world.

## The unique contributions of a journal focused on Israeli health care

In the IJHPR’s inaugural editorial [1], which was published in January 2012, we discussed why we sensed a need for a new journal which would have Israeli health care as its primary focus. With the journal about to celebrate its second birthday (and with this editorial constituting the IJHPR’s 100^th^ article), this is an opportune time to reflect on the extent to which the journal’s experience has validated those intuitions. In the paragraphs that follow we present quotes from the inaugural editorial in **bold** and our current reflections in *italics*.

“First, we wanted to promote a new interdisciplinary synergy between studies of different aspects of Israeli health care where the results of studies done by scholars in a number of health-relevant fields would be gathered in the same publication”.

The journal has clearly succeeded in pulling together contributions from a wide range of fields such as economics, political science, sociology, epidemiology, environmental health, etc. IJHPR articles have also benefited from interdisciplinary synergy through the review process, in which we often involve reviewers from disciplines other than those of the manuscript’s authors. We are also beginning to see new IJHPR articles that build on the findings of previously-published IJHPR articles that are substantively related but written from a different disciplinary perspective; we hope to see more of this as the journal matures.

“Second, we wanted to create a journal in which the publication criteria simultaneously include the contribution to both health systems around the world and to Israeli health care”.

We are pleased to have been able to provide a forum for excellent articles about Israel that also have relevance beyond Israel. In many cases, reviewer comments have helped authors better explicate the international relevance of their studies of developments in Israel. Their implications for health systems around the world have been further elucidated by commentaries by leading international scholars. Some of these “local” articles have even gone on to receive BioMed Central’s “highly accessed” designation, reflecting substantial international readership.

“Third, IJHPR provides us with an opportunity to focus attention on specific areas of particular interest to Israel. Some of these will be areas in which Israel has important accomplishments in the field and/or research… Others will be areas in which Israel is grappling with challenges similar to those in many other countries”.

The IJHPR’s article collections were instituted to take advantage of this opportunity. To date we have launched three article collections: 1) Quality of care in Israel and beyond; 2) The healthcare workforce; and 3) Health promotion and disease prevention. A fourth collection, on prioritization, is due to be launched in early 2014 and the editorial board is prepared to consider proposals for additional article collections.

*Moreover, sometimes individual articles are sufficiently powerful to focus attention – on their own – on a specific area of particular interest to Israel. This would seem to be the case for the 20 IJHPR articles published to date that have been accessed over 2,000 times (see*http://www.ijhpr.org/mostviewed/alltime*).*

“Fourth, we are interested in using IJHPR as a vehicle for engaging scholars from around the world in the health policy development process in Israel”.

The invited commentaries have been the IJHPR’s main vehicles for engaging scholars from around the world, and these have proven to be successful beyond our hopes and expectations. Thus far we have had 7 such contributions from Harvard-based scholars, 5 from scholars at the London School of Hygiene and affiliated institutions, 3 from Johns Hopkins, 2 from Yale, etc. The commentaries have been extremely well-received by the authors on whose works they have commented, as well as by the IJHPR’s readers, and by reviewing bodies such as Thomson Reuters.

For 2014, we are exploring the possibility of building on this success by publishing a few articles by leading international scholars about recent health policy developments in their own countries in areas of particular relevance to Israel. These would then be accompanied by commentaries by Israeli health care thought leaders. The editorial board would welcome suggestions of possible topics, and possible authors, for this experimental venture.

## The year ahead

Other plans and hopes for the year ahead include:

• Continuing to attract, review and publish a steady flow of high-quality original research articles, integrative articles and commentaries

• Publishing a supplement with abstracts from the 2013 Jerusalem International Health Policy Conference

• Sharing, with our international scholarly audience, the eagerly awaited recommendations of the “Advisory Board for Strengthening the Public Health System”

• Publishing articles on topics of particular interest to Israel at this time, such as e-health; the public-private mix in health care; and the changing roles and boundaries of the health care professions

• Organizing occasional symposia which would feature outstanding IJHPR articles and their implications for health policy

• Experimenting with new ways to disseminate our research findings, including those involving social media

## The ongoing opportunities and challenges in cross-national learning

We conclude by reiterating two quotes from the journal’s inaugural commentary [2] by Martin McKee of the London School of Hygiene and Tropical Medicine, as they are as relevant today as they were when initially written:

*“There is enormous scope to learn lessons from health systems in other countries. The challenge is to learn the* ***right*** *lessons. The Israel Journal of Health Policy Research offers an important new platform where researchers and policy makers from different countries can come together to understand each other and share their experiences”.*

“As a regular visitor to Israel, I am aware of the remarkable capacity of the Israeli health research community, its willingness to innovate, and its capacity to learn lessons from elsewhere. Yet lesson learning is not straightforward… It demands that we ask not just “will it work?” but “what are the conditions in which it will work?” and “do those conditions exist in my situation?” …. Above all, it requires a process of enquiry and reflection, ideally one that involves those from elsewhere who can challenge one’s assumptions and ask “why does it have to be so”.

We plan to engage in continuous improvement and to provide an increasingly valuable resource for all those who share the journal’s interest in health policy in Israel and beyond. Accordingly, we welcome your thoughts on how the journal might be even more effective in future years. In particular, in light of Martin McKee’s remarks quoted above, we would welcome your suggestions on how we can best foster cross-national dialogue that is simultaneously vigorous and circumspect.

## Authors’ information

Avi Israeli is the Dr. Julien Rozan Professor of Family Medicine and Health Care at the Hadassah – Hebrew University Medical Center; Director of the Department of Health Policy, Health Care Management and Health Economics, Hebrew University – Hadassah Braun School of Public Health & Community Medicine; Chief Scientist of the Ministry of Health; and co-editor of the IJHPR.

Bruce Rosen is Director of the Smokler Center for Health Policy Research at the Myers-JDC-Brookdale Institute, as well as co-editor of the IJHPR.
